# Gene-specific cell labeling using *MiMIC* transposons

**DOI:** 10.1093/nar/gkv113

**Published:** 2015-02-20

**Authors:** Joshua P. Gnerer, Koen J. T. Venken, Herman A. Dierick

**Affiliations:** 1 Department of Molecular & Human Genetics, Baylor College of Medicine, Houston, TX 77030, USA; 2 Verna and Marrs McLean Department of Biochemistry and Molecular Biology, Baylor College of Medicine, Houston, TX 77030, USA; 3 Department of Pharmacology, Baylor College of Medicine, Houston, TX 77030, USA; 4 Dan L. Ducan Cancer Center, Baylor College of Medicine, Houston, TX 77030, USA; 5 Program in Integrative and Molecular Biomedical Sciences, Baylor College of Medicine, Houston, TX 77030, USA; 6 Program in Developmental Biology, Baylor College of Medicine, Houston, TX 77030, USA; 7 Department of Pathology and Immunology, Baylor College of Medicine, Houston, TX 77030, USA; 8 Department of Neuroscience, Baylor College of Medicine, Houston, TX 77030, USA

## Abstract

Binary expression systems such as GAL4/UAS, LexA/LexAop and QF/QUAS have greatly enhanced the power of *Drosophila* as a model organism by allowing spatio-temporal manipulation of gene function as well as cell and neural circuit function. Tissue-specific expression of these heterologous transcription factors relies on random transposon integration near enhancers or promoters that drive the binary transcription factor embedded in the transposon. Alternatively, gene-specific promoter elements are directly fused to the binary factor within the transposon followed by random or site-specific integration. However, such insertions do not consistently recapitulate endogenous expression. We used *Minos-Mediated Integration Cassette* (*MiMIC*) transposons to convert host loci into reliable gene-specific binary effectors. *MiMIC* transposons allow recombinase-mediated cassette exchange to modify the transposon content. We developed novel exchange cassettes to convert coding intronic *MiMIC* insertions into gene-specific binary factor protein-traps. In addition, we expanded the set of binary factor exchange cassettes available for non-coding intronic *MiMIC* insertions. We show that binary factor conversions of different insertions in the same locus have indistinguishable expression patterns, suggesting that they reliably reflect endogenous gene expression. We show the efficacy and broad applicability of these new tools by dissecting the cellular expression patterns of the *Drosophila* serotonin receptor gene family.

## INTRODUCTION

The continued development of novel molecular genetic technologies has been critical for the staying power of *Drosophila melanogaster* as a model system in biology. The early use of P-element transposons to generate transgenic flies jumpstarted a molecular genetic revolution still ongoing today (1,2). A subsequent technological milestone was the development of the first binary gene expression system that uses the yeast transcription factor GAL4 to activate any gene of interest cloned downstream of the *Upstream Activating Sequence* (*UAS*) (3). Both components of the binary system are integrated separately into a fly's genome through transposon-mediated transgenesis and ‘activated’ by a genetic cross of the two transgenic strains.

The expression pattern of *GAL4* and by extension its target downstream of the *UAS* promoter is either driven by a cloned promoter fragment (promoter-*GAL4*) or by a local enhancer (enhancer-*GAL4*). Promoter-*GAL4*s drive expression of *GAL4* based on a defined promoter fragment cloned into a *GAL4* expression vector, which is typically inserted into the *Drosophila* genome through random transposition. Such promoter-*GAL4* lines do not always accurately reflect endogenous expression of a gene for two reasons. First, the cloned fragment may lack enhancer and/or repressor elements necessary for correct regulation of the gene. Second, the insert may be affected by the genomic context surrounding the integration site (4). Enhancer-*GAL4*s are *GAL4*-containing transposons that express *GAL4* in the pattern of local enhancers in the vicinity of the integration site of the *GAL4*-containing transposon. These lines also do not always accurately recapitulate endogenous expression due to several possible mechanisms, such as size and orientation of the transposon, and distance to the promoter (5).

One strategy to generate gene-specific *GAL4* lines that faithfully reproduce endogenous gene expression is to replace the first coding exon of a gene with a *GAL4* encoding exon through homologous recombination (6–8). This strategy is genetically cumbersome but is somewhat easier when implemented in large genomic fragments that are then inserted into specific predesigned docking sites in the fly genome (9). This type of site-specific integration relies on a viral binary system composed of the bacteriophage ϕC31 integrase and its complementary DNA attachment recognition sites, *attP* and *attB* (10). Once *attP* sites were introduced into the fly genome using transposition embryos injected with integrase and plasmid DNA containing an *attB* site could be efficiently transformed by specific integration into the *attP* sites engineered into the fly's genome.

*Minos-Mediated Integration Cassette* (*MiMIC*) is a specialized transposon that carries two inverted *attP* sites that allow flexible conversion of resident loci through ϕC31 recombinase-mediated cassette exchange (RMCE) (11,12). This transposon contains a dominant body color marker and a stop cassette with a splice acceptor that can mutate a gene when it lands in the right orientation in an intron. Many thousands of *MiMIC* insertions have been generated and are publicly available from the stock center designated as *MI* lines (11,13). What sets this transposon apart from other mutagenic transposons however is that it can be locally modified once inserted in a gene because of the inverted *attP* sites so that the content of the transposon can be exchanged with a new cassette allowing limitless modification of the locus (11). Two examples of the versatility of this transposon system are protein- and gene-traps. A protein-trap is made by converting a *MiMIC* insertion in a coding intron into an artificial exon encoding a protein tag (e.g. superfolder Green Fluorescent Protein (GFP)) to visualize endogenous protein localization. A gene-trap, in contrast, is based on conversion of 5′ non-coding intronic insertions into an artificial terminal exon. Such insertions can be used to document the endogenous cellular expression pattern of a host gene when a binary factor (e.g. GAL4) is inserted (11), but only ∼13% of *MiMIC* insertions are located in 5′ non-coding introns. That means that this strategy is not feasible for ∼87% of *MiMIC* insertions (11). In order to make all intragenic intronic insertions available for conversion (∼46% of all *MiMIC* insertions) (11), we designed a set of protein-trap cassettes for the conversion of coding intronic *MiMIC* insertions into gene-specific binary factors. In addition, we created three new gene-trap cassettes for the binary transcription factor LexA (14), the drug-inducible transcription factor GeneSwitch (15,16) and GAL80 (17), a negative regulator of GAL4. We have tested these novel protein- and gene-trap conversion cassettes on 16 different *MiMIC* insertions in 10 different genes and show that this conversion strategy reliably reflects endogenous gene expression. These novel tools will be useful for gene-specific manipulations of gene function as well as cell and neural circuit function.

## MATERIALS AND METHODS

### Fly stocks

The following fly stocks were obtained from the Bloomington Stock Center: y^1^ w*; Mi{MIC}5-HT1A^MI01140^ (BL43553), y^1^ w*; Mi{MIC}5-HT1A^MI04464^ (BL37456), y^1^ w*; Mi{MIC}5-HT1B^MI05213^ (BL41063), y^1^ w*; Mi{MIC}5-HT2A^MI00459^/TM6, Tb^1^ (BL31012), y^1^ w*; Mi{MIC}5-HT2A^MI03299^ (BL31177), y^1^ w*; Mi{MIC}5-HT2B^MI05208^/TM3, Sb^1^ Ser^1^ (BL42994), y^1^ w*; Mi{MIC}5-HT2B^MI07403^ (BL43706), y^1^ w*; Mi{MIC}5-HT2B^MI06500^ (BL40810), y^1^ w*; Mi{MIC}5-HT7^MI00215^/TM6B, Tb^1^ (BL30667), y^1^ Mi{MIC}arm^MI08675^ w* (BL44994), y^1^ w*; Mi{MIC}Vmat^MI07680^/SM6a (BL43752), y^1^ w*; Mi{MIC}Ubp64E^MI01350^/TM3, Sb^1^ Ser^1^ (BL35943), y^1^ w*; Mi{MIC}gfA^MI07507^ (BL43836), y^1^ w*; Mi{MIC}ITP^MI00349^ CG4622^MI00349^ (BL30713), P{26XLexAop2-mCD8::GFP}attP2 (BL32207). The following MiMIC lines were a gift from Hugo J. Bellen (Baylor College of Medicine): y^1^ w*; Mi{MIC}5-HT1A^MI01468^ and y^1^ w*; Mi{MIC}5-HT2B^MI03466^. The 5-HT1A and 5-HT7 promoter-GAL4 lines were a gift from Charles Nichols (Louisiana State University Health Sciences Center). The 10xUAS-IVS-syn21-GFPp10 reporter (18) flies were a gift from Barret Pfeiffer and Gerry Rubin (Janelia Farm Research Campus).

### Construction of the protein-trap and gene-trap cassettes

Protein-trap cassettes with T2A fused to binary factors were made by cloning polymerase chain reaction (PCR) fragments into the previously generated protein-trap vectors for the three different intron phases: pBS-KS-attB1–2PT-SA-SD-0, 1 or 2 (11). To generate the *T2A-GAL4* plasmid, we amplified the *GAL4* sequence from the GAL4 gene-trap cassette by using a forward primer matching the first 22 bp of the *GAL4* sequence connected to the T2A sequence and a *Bam*HI site and a reverse primer to the Hsp70 3′UTR sequence followed by a *Bam*HI site. The resulting PCR product was digested with *Bam*HI and cloned into the *Bam*HI digested protein-trap cassettes for all three frames. All clones were verified by sequencing. The same procedure was used to generate *T2A-GeneSwitch, T2A-LexA* and *T2A-GAL80* plasmids. To generate the corresponding gene-trap plasmids, the same strategy was used but the fragments were cloned into the gene-trap vector, pBS-KS-attB1–2-GT-SA (11) (Supplementary Figure S1).

### ϕC31 integrase-mediated RMCE

All the genes and alleles that were used in the conversion experiments are listed in Table 1 and Supplementary Figure S2. Conversions were performed as previously described (11). Briefly, we injected plasmid DNA of the above-described exchange cassettes into fertilized embryos (before they were cellularized) that were derived from flies with each *MiMIC* insertion crossed to flies with a ϕC31 integrase source on the X chromosome and an appropriate balancer chromosome (i.e. for chromosome 2 or 3) so that the *MiMIC* insertion remains balanced during the conversion process and that successful conversion events can be recovered by scoring for the absence of the *yellow^+^* (*y^+^*) dominant body color marker. Adults that emerged after injection were crossed to *y w* stocks with the appropriate balancer and *y^−^* offspring from these crosses were selected to establish a new stock with the successful conversion event. These flies were subsequently analyzed molecularly to verify correct integration of the exchange cassettes.

**Table 1.** Genes, *MiMIC* alleles and conversions

					Conversions				
*Gene*	*CG*	*MiMIC*	PT/GT	Phase	G4	GFP	G80	GS	LA
*5-HT1A*	*CG16720*	*MI01140*	PT	2	+	∼		-	-
		*MI01468*	PT	2	+				
		*MI04464*	PT	2	+	∼			
*5-HT1B*	*CG15113*	*MI05213*	PT	0	+	-		-	-
*5-HT2A*	*CG1056*	*MI00459*	PT	2	+	-	+	-	-
		*MI03299*	GT		+		+	+	-
*5-HT2B*	*CG42796*	*MI05208*	PT	1	+	-	+		
		*MI06500*	PT	1	+		+		
		*MI03466*	GT		+		+	+	
		*MI07403*	GT		+		+	+	+
*5-HT7*	*CG12073*	*MI00215*	GT		+		∼	+	+
*arm*	*CG11579*	*MI08675*	PT	1	+	+	+		
*Vmat*	*CG33528*	*MI07680*	PT	0	+	+			
*Ubp64E*	*CG5486*	*MI01350*	GT		+		+		
*nAchRa7*	*CG32538*	*MI07507*	GT		+				
*Itp*	*CG4622*	*MI00349*	GT		+				
			Protein-Trap		100%	29%	100%	0%	0%
			Gene-Trap		100%	NA	80%	100%	67%

List of all the genes and alleles converted with the different gene-trap and protein-trap cassettes described in this manuscript. All 16 alleles converted with *GAL4* (*G4*) in protein- and/or gene-trap configuration showed reproducible and internally consistent expression patterns when crossed to *UAS-GFP. GAL4* (*G4*) and *GAL80* (*G80*) conversions were most successful and worked reliably in both protein-trap and gene-trap configurations. *EGFP* cassette conversions showed only strong expression in two of the seven conversions that we attempted. *GeneSwitch* (*GS*) conversions worked reliably in the gene-trap configuration (all four) but not in the protein-trap configuration. *LexA* (*LA*) conversions only worked for some gene-traps and not for protein-traps.

### Molecular characterization of the conversion events

The conversion cassettes can recombine into the locus in a forward or reverse orientation relative to the direction of the locus and require screening of the integration orientation. PCR-based verification of RMCE events was performed as previously described (11). Briefly, DNA was extracted from a small number of adult flies using the PureLink Genomic DNA Mini kit (Life Technologies) and PCR was performed with cassette-specific primers and *MiMIC*-specific primers to determine the orientation of the conversion event. The primers that were used for PCR confirmation of conversion events are listed in Supplementary Table S1. PCR conditions for the conversion events were as follows: denaturation at 94°C for 10 min, 40 cycles at 94°C for 30 s, 60°C for 30 s and 72°C for 60 s and post amplification extension at 72°C for 10 min.

### Expression analysis of the gene-specific binary factor conversion strains

Flies with confirmed conversion events were crossed to *10xUAS-IVS-syn10-GFPp10* (18). Staining and imaging was performed as previously described (19) with the following modifications. Adult brains were dissected and fixed in ice-cold 4% paraformaldehyde–phosphate-buffered saline (PBS) for a total of 1 h. Next, the brains were rinsed two times with PBS-0.5% Triton X-100 (PBT) and then washed twice for 30 min in PBT at room temperature. The brains were then blocked in 5% normal goat serum (NGS) in PBT for 1 h at room temperature. Samples were incubated in 5% NGS/PBT with primary antibody for 48 h at 4°C. After two 30 min washes with PBT, the brains were incubated in 5% NGS/PBT with secondary antibody for 48 h at 4°C. The brains were then washed two times for 30 min at room temperature and then for two days at 4°C. Finally, brains were mounted in SlowFade mounting medium (Invitrogen) and covered with a no. 0 glass coverslip that was separated from the slide by two strips of scotch tape. Immunostained brains were imaged with an inverted Zeiss Confocal Microscope (Axioplan 2) equipped with an ApoTome (Zeiss). The following primary antibodies were used for immunofluorescence: mouse anti-Dlg (1:100; Developmental Studies Hybridoma Bank); rabbit anti-GFP (1:500; Invitrogen) or mouse anti-GFP (1:200; NeuroMab); rabbit ds-Red (1:200; Clonetech). Alexafluor secondary antibodies were obtained from Invitrogen and were used at a final concentration of 1:500. Images were analyzed with AxioVision Software (Version 4.8.2.0 Zeiss).

## RESULTS AND DISCUSSION

### A novel T2A-based GAL4 exchange cassette for RMCE

**Figure 1.**
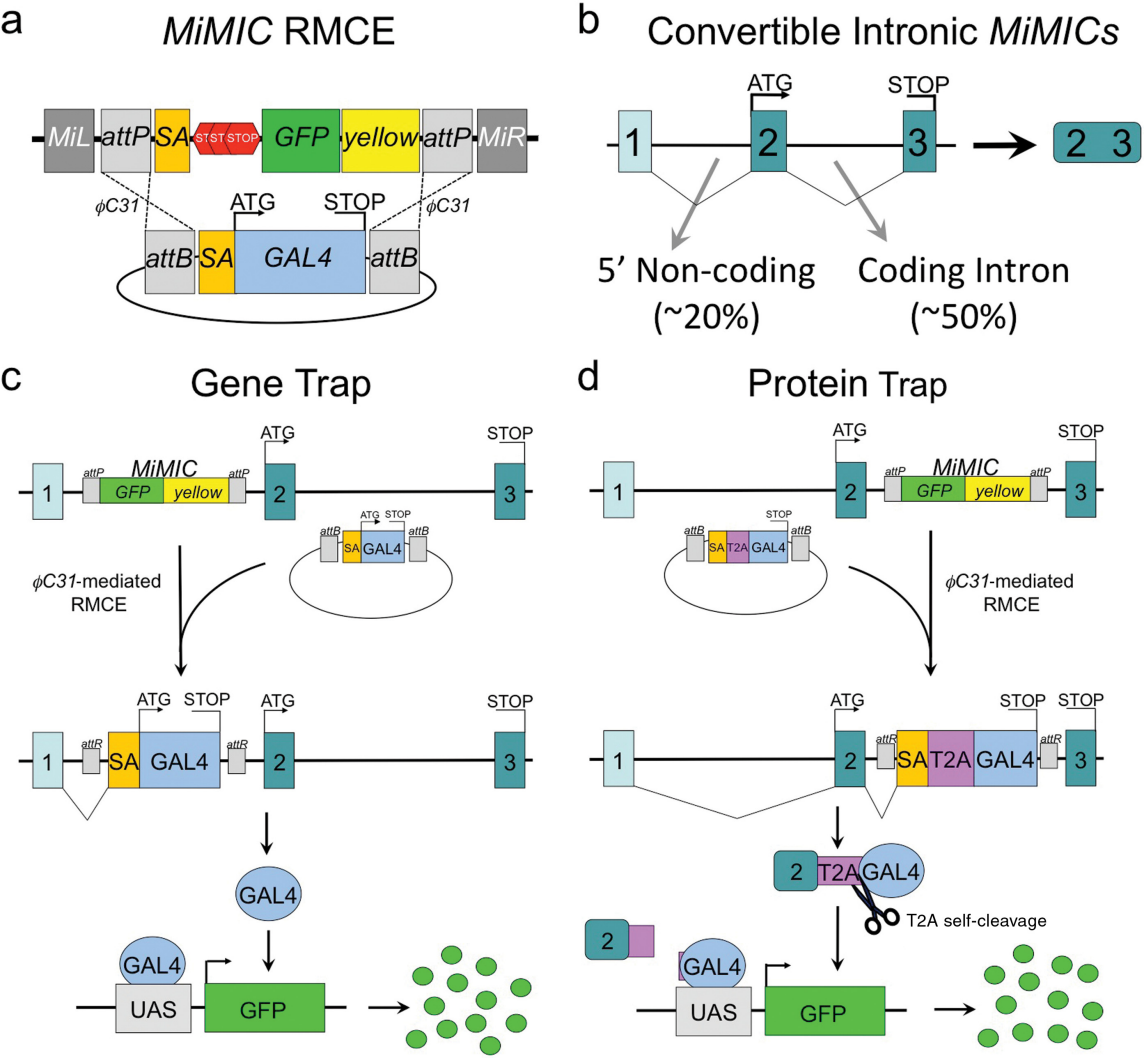
GAL4 gene-trap and protein-trap *MiMIC* conversion strategy for expression analysis. (**a**) Schematic of ϕC31-mediated RMCE to swap the content of a *MiMIC* transposon with a GAL4 gene-trap. Recombination can occur in reverse or forward orientation relative to the targeted locus and is shown here in the forward orientation. (**b**) Gene diagram of a hypothetical 3 exon gene with a 5′ non-coding and coding intron showing the distribution of convertible intronic *MiMIC* insertions. (**c**) Schematic of gene-trap GAL4 conversion between a 5′ non-coding intronic *MiMIC* insertion and a splice acceptor GAL4 cassette. (**d**) Schematic of a protein-trap conversion strategy using a coding intronic *MiMIC* insertion and splice acceptor T2A-GAL4 cassette. RMCE, recombinase-mediated cassette exchange; *MiMIC, Minos-Mediated Integration Cassette*.

*MiMIC* insertions contain two inverted *attP* sites that allow swapping of the transposon content using ϕC31-mediated RMCE (Figure 1a) (11). To expand the existing GAL4 conversion of 5′ non-coding intronic insertions (∼20% of all intragenic *MiMIC* insertions), to include coding intronic *MiMIC* insertions (∼50% of the intragenic *MiMIC* insertions) (Figure 1b), we designed a novel exchange cassette that contains a splice acceptor followed by a self-cleaving *T2A* peptide sequence fused to the GAL4 coding sequence (20) ending in a stop codon and stabilizing 3′UTR (Supplementary Figure S1). We made conversion cassettes for all three frames to accommodate conversion of any coding intronic insertion regardless of the intron phase. A gene-trap cassette in a 5′ non-coding intron is spliced onto the upstream non-coding exon of the host gene and leads to translation of GAL4, which can then activate an effector gene downstream of the *UAS* promoter (Figure 1c). In contrast, the newly designed protein-trap cassette splices to the upstream coding exon fusing the GAL4 gene sequence to a piece of the host gene separated by the T2A sequence. During translation of this hybrid mRNA, the GAL4 protein part is released due to failure of peptide-bond synthesis at the last codon of the T2A ‘self-cleaving’ peptide (21). This generates a truncated version of the native protein attached to T2A and a GAL4 transcription factor molecule that can mark the cellular expression pattern of the host gene through activation of a *UAS*-reporter (Figure 1d). The C-terminal Proline of the T2A peptide will become the first amino acid of GAL4.

### T2A-GAL4 conversion of 5-HT1A and 5-HT1B express in different mushroom body lobes

**Figure 2.**
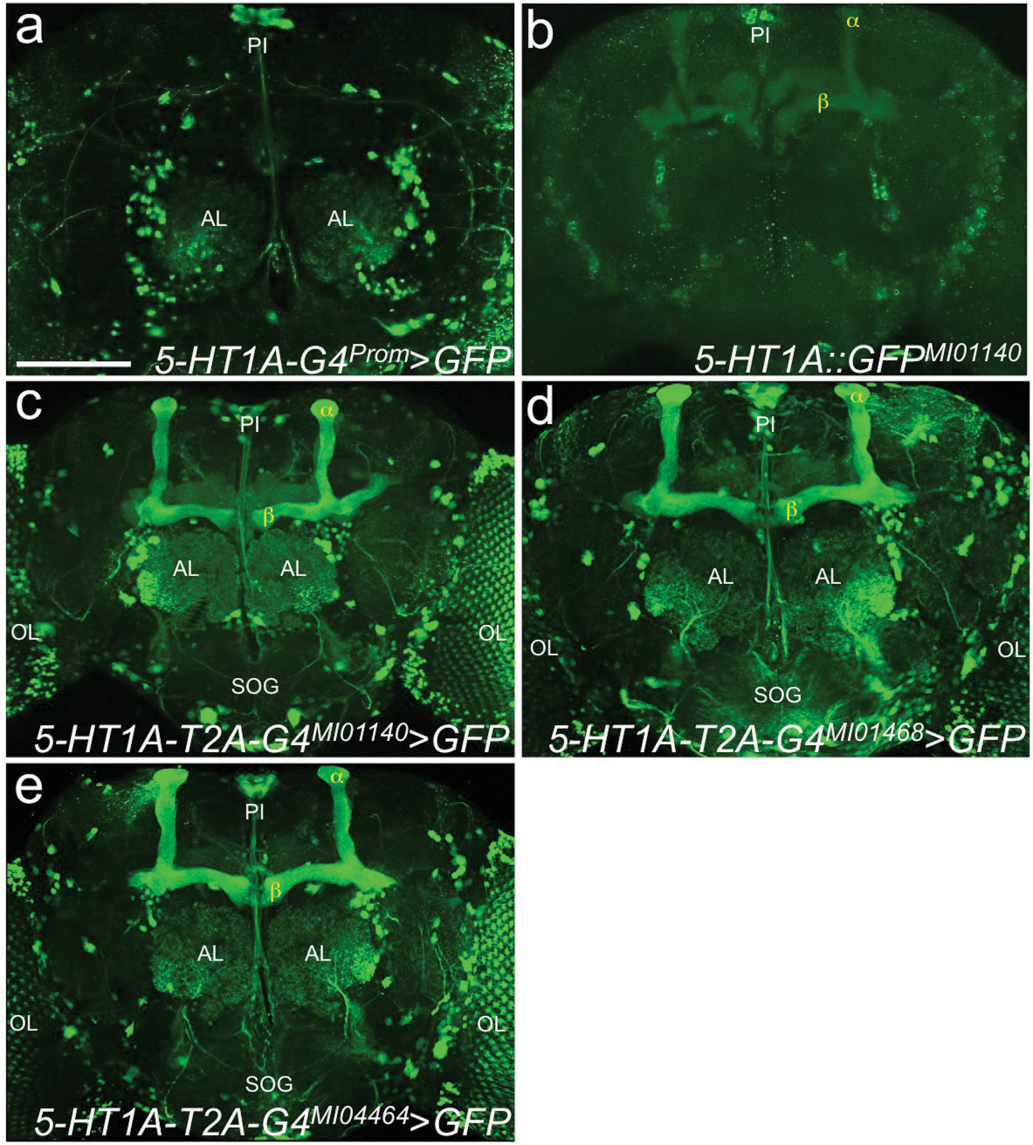
Protein fusions between 5-HT1A and T2A-GAL4 produce robust, reproducible and complete expression patterns. (**a**) A 5kb 5-HT1A promoter fragment fused to GAL4 (*5-HT1A-GAL4^Prom^*) drives strong expression of *UAS-GFP* in the *PI* and AL. (**b**) An internally tagged EGFP protein fusion of 5-HT1A (*5-HT1A::EGFP^MI01140^*) shows faint expression in the MBs, *PI*, antennal neurons and SOG. (**c**) On the other hand, a protein fusion between 5-HT1A and T2A-GAL4 (*5-HT1A-T2A-GAL4^MI01140^*) drives expression of *UAS-GFP* in *PI*, AL, OL, SOG and MB (most prominently in α and β lobes). (**d–e**) Different protein fusions between 5-HT1A and T2A-GAL4, *5-HT1A-T2A-GAL4^MI01468^* (d) and *5-HT1A-T2A-GAL4^MI04464^* (e) show very similar expression patterns as *5-HT1A-T2A-GAL4^MI01140^* (c). Scale bar, 100 μm. MB, mushroom bodies; *PI, pars intercerebralis*; AL, antennal lobes; OL, optic lobes; SOG, suboesophageal ganglion.

To test the new conversion strategy, we first focused on the 5-HT1A receptor, one of the five *Drosophila* serotonin receptors (13,22–24). Currently, antibodies against this (and most of the other) 5-HT receptors are lacking and most of our knowledge regarding receptor expression patterns is derived from enhancer- or promoter-*GAL4s* (25–29). Such GAL4 lines do not always accurately reflect endogenous expression. Indeed, when we stained adult brains of flies expressing *UAS-GFP* (18) driven by a *5-HT1A* promoter-*GAL4* (*5-HT1A-GAL4^Prom^* (30)) (Supplementary Figure S2a), we observed no detectable expression in the mushroom bodies (Figure 2a), a brain region previously shown to express *5-HT1A* mRNA (27) and where the 5-HT1A receptor is functionally required for sleep (27) and anesthesia resistant memory (31). To investigate this discrepancy, we first examined a coding intronic *MiMIC* insertion (*Mi{MIC}5-HT1A::EGFP^MI01140^*) (Supplementary Figure S2a), in which a GFP exon was integrated in frame within the 5-HT1A locus through RMCE (11), effectively GFP-tagging the 5-HT1A protein. When we stained brains from homozygous *Mi{MIC}5-HT1A::EGFP^MI01140^* flies, we observed faint expression in the mushroom body in addition to scattered GFP expressing cells throughout the protocerebrum including the *pars intercerebralis* (*PI*) (Figure 2b).

We next converted the same coding intronic *MiMIC* insertion in the 5-HT1A locus (Supplementary Figure S2a) with the newly designed *T2A-GAL4* exchange cassette. Adult brains from *Mi{MIC}5-HT1A::GAL4^MI01140^* animals crossed to *UAS-GFP* show prominent signal in the mushroom bodies (α and β lobes) (Figure 2c and Supplementary Video 1) consistent with both *in situ* hybridization data (27) and the functional requirement of 5-HT1A in the mushroom bodies (27,31). *T2A-GAL4* conversion of two additional *MiMIC* alleles in the *5-HT1A* locus (*Mi{MIC}5-HT1A::GAL4^MI04464^* and *Mi{MIC}5-HT1A::GAL4^MI01468^* Supplementary Figure S2a) shows the same pattern of expression (Figure 2d and e).

**Figure 3.**
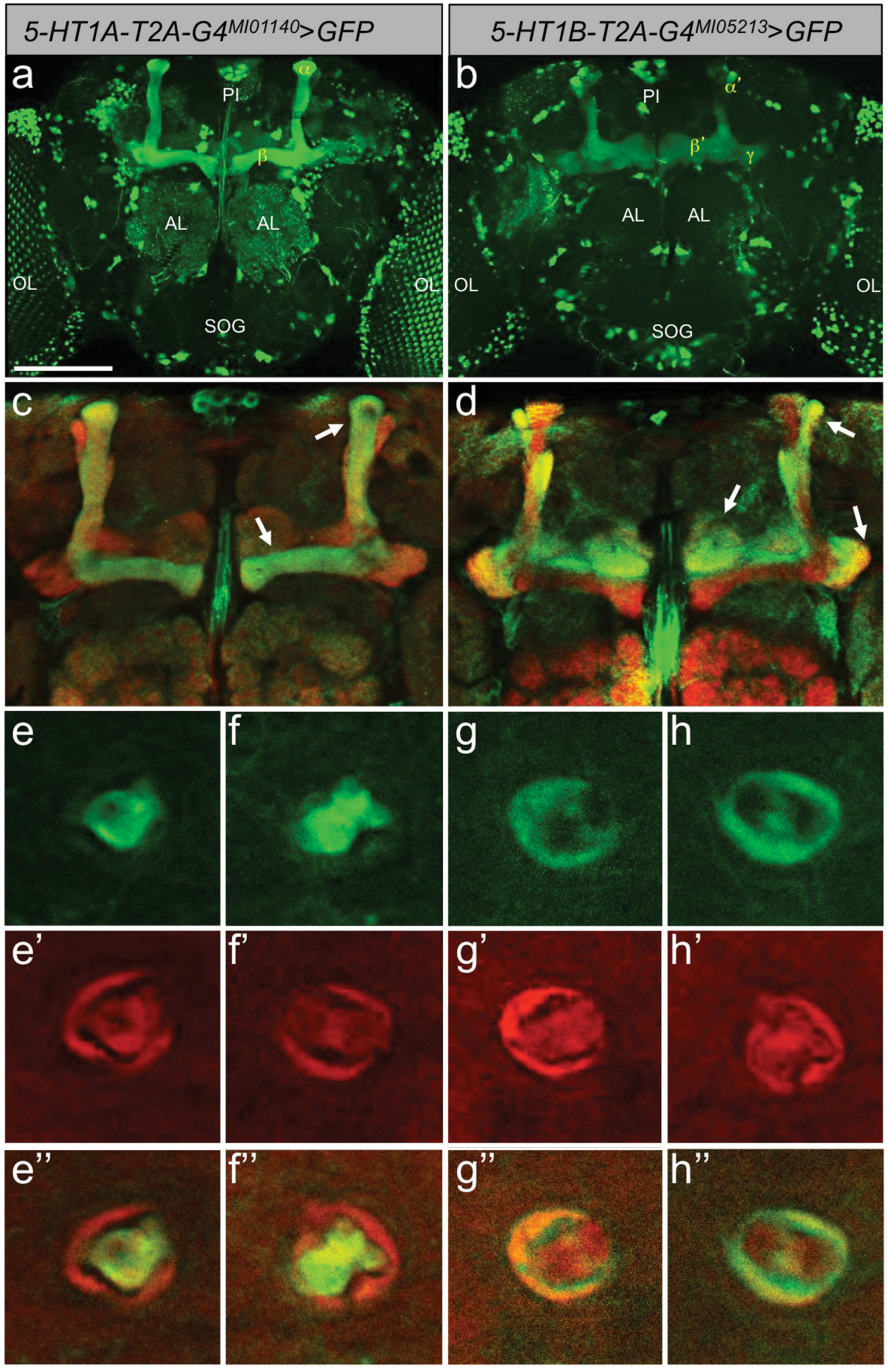
5-HT1A and 5-HT1B T2A-GAL4 protein-traps uncover differential MB expression. (**a**) A protein-trap event between 5-HT1A and T2A-GAL4 (*5-HT1A-T2A-GAL4^MI01140^*) drives expression of *UAS-GFP* in *PI*, AL, OL, SOG and MB (most prominently in the α and β lobes, Supplementary Video 1, see also Figure 2). (**b**) A protein-trap event between 5-HT1B and T2A-GAL4 (*5-HT1B-T2A-GAL4^MI05213^*) also drives expression of *UAS-GFP* in *PI*, AL, OL, SOG and MB, but more prominently in the α’ and β’ and γ lobes. (**c**) GFP staining pattern (in green) of the *Mi{MIC}5-HT1A-T2A-GAL4^MI01140^* driving expression of *5xUAS-mCD8::GFP* zoomed in on the mushroom bodies (MBs) shows distinct expression in α and β lobes (arrows). Neuropil is stained with an antibody against Dlg, showing the outline of the entire MB in red. (**d**) Same area of the brain as in (c) showing GFP staining pattern of the *Mi{MIC}5-HT1B-T2A-GAL4^MI05213^* driving expression of the same reporter but with distinct expression in α’ and β’ and γ lobes (arrows highlight different lobes in the MB, right-most arrow shows the heel of the MB, indicating γ lobes). GFP staining in the α and β lobes is almost completely excluded. (**e–f**) A slice through the peduncle shows GFP expression of the core α and β lobes of the peduncle in *Mi{MIC}5-HT1A-T2A-GAL4^MI01140^*. (**g–h**) Image taken at the same level in the peduncle shows the outer layer stained in *Mi{MIC}5-HT1B-T2A-GAL4^MI05213^* indicating expression in the γ lobes. (**e’–h’**) The same images as in (e-h) showing Dlg expression. (**e”–h”**) The same images as in (e–h) showing merged green (GFP) and red (Dlg) channels. MB, mushroom bodies; *PI, pars intercerebralis*; AL, antennal lobes; OL, optic lobes; SOG, suboesophageal ganglion.

To further test the efficacy of the *T2A-GAL4* cassette, we converted the single coding intronic *MiMIC* insertion in the *5-HT1B* locus (Supplementary Figure S2b). As for *Mi{MIC}5-HT1A::GAL4^MI01140^* (Figure 3a), we found strong adult brain expression of *GFP* with *Mi{MIC}5-HT1B-GAL4^MI05213^* driving the same *UAS-GFP* reporter (18) (Figure 3b). Previously a promoter-*GAL4* line for 5-HT1B also showed strong expression in the mushroom bodies consistent with antibody staining for this receptor (25). Our T2A-GAL4 converted 5-HT1B also shows expression in the mushroom bodies. Mushroom body expression in the two 5-HT1 class receptors is distinct however, with 5-HT1A predominantly staining α and β lobes, while 5-HT1B predominantly staining α’, β’ and γ lobes (32) (Figure 3c–h”, Supplementary Video 2).

### Gene- and protein-trap GAL4 conversions in the same gene produce similar patterns

**Figure 4.**
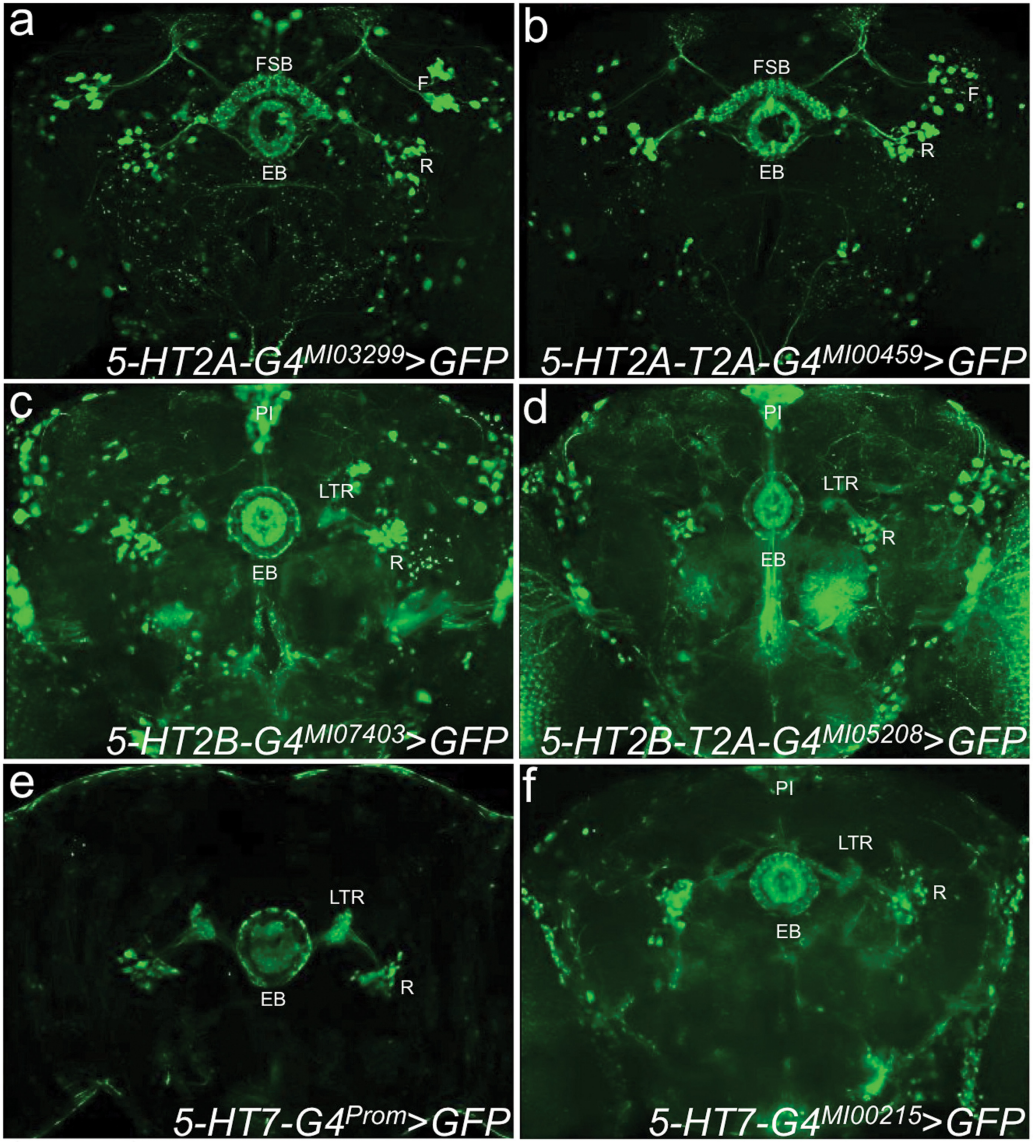
T2A-GAL4 and gene-trap GAL4 conversions reveal very similar expression patterns. (**a**) A gene-trap event between 5-HT2A and GAL4 (*5-HT2A-GAL4^MI03299^*) drives expression of *UAS-GFP* in EB, R-cells, dorsal FSB, F-cells with dendritic tufts in dorsal protocrebrum. (**b**) A protein-trap event between 5-HT2A and T2A-GAL4 (*5-HT2A-T2A-GAL4^MI00459^*) driving expression of *UAS-GFP* shows an expression pattern very similar to *5-HT2A-T2A-GAL4^MI00459^* expression (Supplementary Video 3). (**c**) A gene-trap event between 5-HT2B and GAL4 (*5-HT2B-GAL4^MI07403^*) drives expression of *UAS-GFP* in *PI*, EB, R-cells (Supplementary Figure S3). (**d**) A protein-trap event between 5-HT2B and T2A-GAL4 (*5-HT2B-T2A-GAL4^MI05208^*) driving expression of *UAS-GFP* shows an expression pattern highly similar to *5-HT2B-T2A-GAL4^MI05208^* expression (Supplementary Figure S3). (**e**) The 5-HT7 promoter fragment fused to GAL4 (*5-HT7-GAL4^Prom^*) drives expression of *UAS-GFP* in EB (outer ring), LTR and R-cells. (**f**) A gene-trap event between 5-HT7 and GAL4 (*5-HT7-GAL4^MI00215^*) drives expression of *UAS-GFP* in a similar pattern to *5-HT7-GAL4^Prom^*, but also includes *PI* and glial staining. *PI, pars intercerebralis*; AL, antennal lobes; OL, optic lobes; MB, mushroom body; EB, ellipsoid body; FSB, fan-shaped body; LTR, lateral triangle; R, neuron ring neuron; F, neuron fan-shaped body neurons of the FSB; SOG, subesophageal ganglion. Scale bar, 100 μm.

We next tested whether the *T2A-GAL4* protein-trap conversions generate the same expression pattern as gene-trap *GAL4* cassettes (11). To do so, we compared the two types of conversion events for *5-HT2A* (Supplementary Figure S2c). Gene-trap and protein-trap *GAL4* conversion of both *5-HT2A MiMIC* insertions (*Mi{MIC}5-HT2A^MI00459^* and *Mi{MIC}5-HT2A^MI03299^*) produced very similar expression patterns, staining the ellipsoid body (EB), lateral triangle with the associated R-cells, the dorsal fan-shaped body (FSB) and the F-cells with characteristic dendritic tufts radiating to the top of the dorsal protocerebrum (33) (Figure 4a–b and Supplementary Video 3). This pattern of expression is distinct from the enhancer trap insertion-based pattern, which showed expression in the EB but not in the FSB, with the caveat that the expression pattern was generated with a *LacZ* reporter (26). Our results confirm that enhancer trap lines do not necessarily recapitulate endogenous expression accurately (5). We next tested the two non-coding and two coding intronic *MiMIC* insertions in the *5-HT2B* locus (Supplementary Figure S2d). All four produced very similar expression patterns regardless of whether we converted a gene-trap or protein-trap, staining strongly in the EB, LTR, R cells and *PI* (Figure 4c–d and Supplementary Figure S3). The consistency of the expression patterns generated by *GAL4* conversion of different *MiMIC* insertions in the same locus (separated by as much as 40 kb) strongly suggests that they faithfully recapitulate the full expression pattern of the host gene.

To complete the conversions of all the 5-HT receptor encoding genes, we converted the single gene-trap *MiMIC* allele in the 5-HT7 locus (*Mi{MIC}5-HT7^MI00215^*) and found that it is expressed in the EB and R-cells similar to the pattern of the promoter-based *GAL4* (*5-HT7-GAL4^Prom^* (28)) (Figure 4e–f and Supplementary Figure S2e).

To further compare the efficacy of T2A-GAL4 protein-traps with those of the GFP protein-traps, we made GFP protein-traps for three coding intronic *MiMIC* insertions in *5-HT1B* (*Mi{MIC}5-HT1B^MI05213^*), *5-HT2A* (*Mi{MIC}5-HT2A^MI0459^*) and *5-HT2B* (*Mi{MIC}5-HT2B^MI05208^*), as we did for *5-HT1A* (Figure 2b). None of the conversions show detectable GFP signal even when stained with antibodies against GFP (data not shown, summarized in Table 1).

Together these results suggest that *GAL4* conversions of both coding and non-coding intronic *MiMIC* insertions can be used as an alternative method to identify the cellular expression pattern of genes for which no antibodies exist and for which GFP protein-traps do not produce visible patterns or only weak expression patterns. While these GAL4 conversions do not provide information on the sub-cellular localization of the endogenous protein, they do make functional manipulation of the neurons possible.

### GAL4 conversions recapitulate expression of GFP-tagged proteins

**Figure 5.**
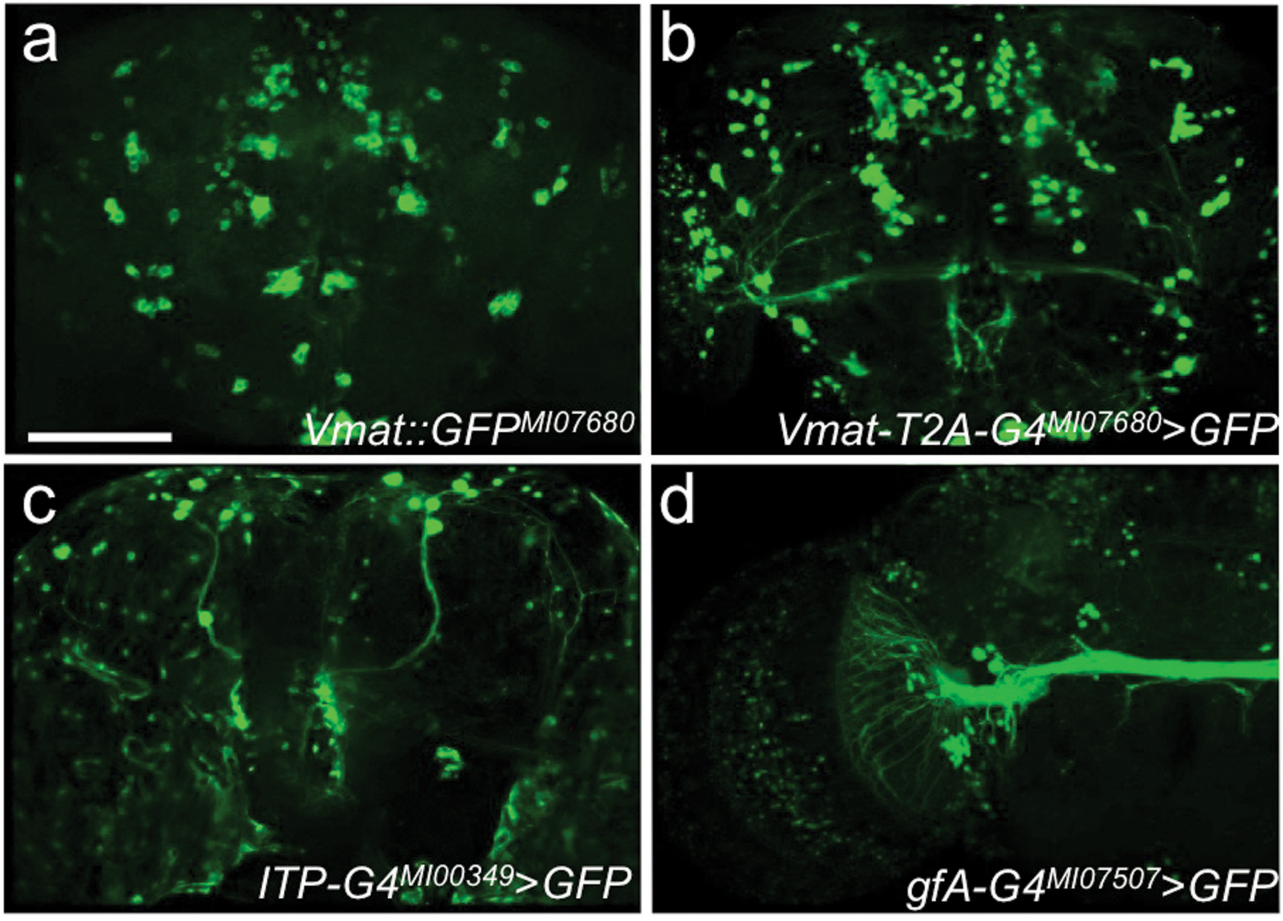
Conversions of genes with known expression patterns. (**a**) An internally tagged EGFP protein fusion of *Vmat* (*Vmat::EGFP^MI07680^*) illustrates scattered labeling of cells throughout the brain similar to its known expression. (**b**) A protein-trap event between Vmat and T2A-GAL4 (*Vmat-T2A-GAL4^MI07680^*) drives similar albeit stronger expression of *UAS-GFP*. (**c**) A gene-trap event between ITP and GAL4 (*ITP-GAL4^MI00349^*) shows expression in clock neurons and also shows expression in glial cells around the brain (Supplementary Video 4). (**d**) A gene-trap event between gfA and GAL4 (*gfA-GAL4^MI07507^*) drives strong expression of *UAS-GFP* in the giant fiber receiving signals from the optic lobe. This nicotinic acetylcholine receptor also strongly expresses in the γ lobes of the MB (Supplementary Video 5). Scale bar, 100 μm.

To further test how accurately these constructs capture the expression patterns of the native genes, we converted several *MiMIC* insertions in genes for which we did succeed in GFP-tagging the protein-traps. *GAL4* conversions of *MiMIC* insertions in *armadillo* (*Mi{MIC}arm^MI08675^*) and *Vesicular monomamine transporter* (*Mi{MIC}Vmat^MI07680^*) show very similar patterns compared to the GFP-tagged proteins (Figure 5a–b and Supplementary Figure S4a–b, Table 1, Supplementary Figure S2f–g). In addition, *GAL4* conversion of *MiMIC* insertions in *Ion transport peptide* (*Mi{MIC}ITP^MI00349^*), *nicotinic Acetylcholine Receptor α7* (*Mi{MIC}gfA^MI07567^*) and *Ubiquitin specific protease 64E* (*Mi{MIC}Ubp64E^MI01350^*) produced patterns consistent with the known expression patterns for these genes (34–36) (Figure 5c–d and data not shown, Supplementary Figure S2h–j and Supplementary Videos 4, 5). These data provide strong evidence that the *MiMIC*-based *GAL4* conversion patterns faithfully represent the expression patterns of the endogenous locus. Further support for this conclusion is the observation that lines derived from different insertions in the same gene have expression patterns that are in many cases indistinguishable from each other, but different from those of promoter-based *GAL4* or enhancer trap lines in those genes. This is likely the case because *MiMIC* conversion lines are generated in the context of the endogenous transcription unit.

### Geneswitch, LexA and GAL80 exchange cassettes

**Figure 6.**
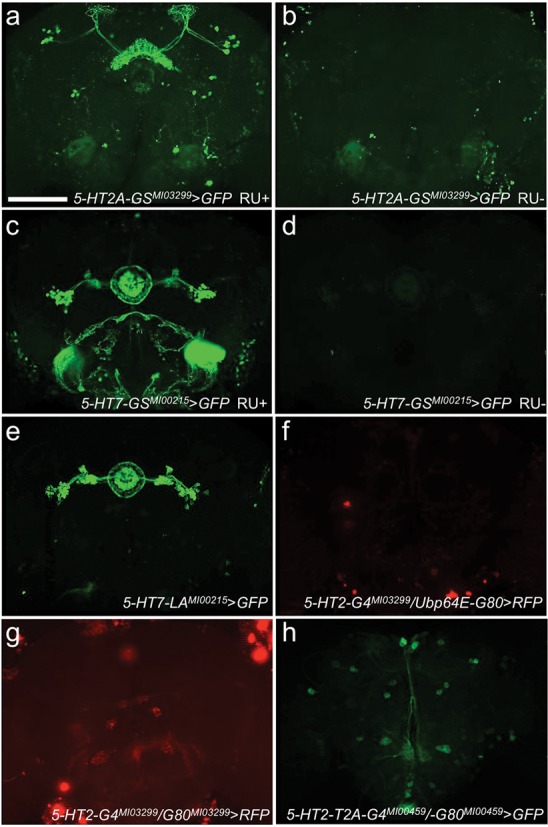
Gene fusions with GeneSwitch, LexA and GAL80. (**a**) A gene-trap event between 5-HT2A and GeneSwtich (*5-HT2A-GS^MI03299^*) drives expression of *UAS-GFP* in a pattern very similar to *5-HT2A-GAL4^MI03299^* driven expression of *UAS-GFP* (Figure 4a), but only after induction with RU486. (**b**) The same insertion (*5-HT2A-GS^MI03299^*>*UAS-GFP*) shows no expression without induction with RU486. (**c**) A gene-trap event between 5-HT7 and GeneSwtich (*5-HT7-GS^MI00215^*) drives strong expression of *UAS-GFP* in EB (and outer ring), LTR and R-cells, very similar to the GAL4 line (*5-HT7-GAL4^MI00215^*) as well as expression in sheath cells surrounding the SOG, only when induced with RU486. (**d**) The same insertion (*5-HT7-GS^MI00215^*) shows very faint expression in EB without RU486. (**e**) A gene-trap event between 5-HT7 and LexA (*5-HT7-LA^MI00215^*) drives expression of *LexAop-mCD8::GFP* exclusively in EB, LTR and R-cells, more similar to the restricted pattern seen in *the promoter fusion* (Figure 4e). (**f**) A gene-trap event between 5-HT2A and GAL4 (*5-HT2A-GAL4^MI03299^*) driving the expression of *UAS-RFP* is completely repressed by co-expression of a gene-trap event between Ubp64e and GAL80 (*Ubp64E-GAL80^MI01350^*) (remaining signal is background noise due to increased gain to better show repression). (**g**) A gene-trap event between 5-HT2A and GAL4 (*5-HT2A-GAL4^MI03299^*) driving the expression of *UAS-RFP* is mostly repressed by co-expression of a GAL80 conversion in the same *MiMIC* insertion (*5-HT2A-GAL80^MI03299^*). (**h**) A protein-trap event between 5-HT2A and GAL4 (*5-HT2A-T2A-GAL4^MI00459^*) driving the expression of *UAS-GFP* is repressed by co-expression of a GAL80 conversion in the same *MiMIC* insertion (*5-HT2A-T2A-GAL80^MI00459^*), except for some expression in the *PI* neurons. *PI, pars intercerebralis*; EB, ellipsoid body; LTR, lateral triangle; R, neuron ring neuron. Scale bar, 100 μm.

We next created three new binary factor gene-trap cassettes: a GeneSwitch (GS) (15,16) cassette for locus-specific inducible expression, a LexA (LA) (14,37) cassette for intersectional (i.e. overlapping) expression and a GAL80 (17,38) cassette allowing cell-specific inhibition through GAL4 repression (39). The expression pattern of the *GS* conversion in the *5-HT2A* locus (*Mi{MIC}5-HT2A^MI03299^*) showed inducible expression indistinguishable from the *GAL4*-mediated conversions (Figure 6a and b). Similarly, the *5-HT7* gene-trap converted with *GS* produced strong inducible expression (Figure 6c and d). We then tested *LA* conversions in the *5-HT2A* and *5-HT7* loci. While the *LA* conversion of *5-HT7* showed strong expression in the EB and R-cells (albeit weaker than the *GAL4* and inducible *GS* patterns) (Figure 6e), the *LA* conversion of *5-HT2A* had little to no staining (data not shown). The lack of *5-HT2A-LA* expression and the weaker *5-HT7-LA* expression that resembles *5-HT7-GAL4^Prom^* expression (Figure 4e) suggest that the *LA* construct is weaker than *GAL4* and *GS*. We next tested whether *GAL80* conversions in the *5-HT2A* and broadly expressing *Ubp64E* genes could block expression from the *5-HT2A-GAL4* conversions. Both mostly inhibited *GAL4* expression (Figure 6f and g). Finally, we created *T2A* protein-trap cassettes for the same binary factors (*T2A-GS, T2A-LA* and *T2A-GAL80*). Like the *GAL80* gene-trap conversions, *T2A-GAL80* conversion of *5-HT2A* inhibited *5-HT2A*-*T2A-GAL4* expression although it did not completely block expression in the *PI* neurons (Figure 6h). However, neither the *T2A-GS* nor the *T2A-LA* constructs showed detectable expression in any of the loci that we tested (data not shown, summarized in Table 1), suggesting that peptide bond skipping may be ineffective in these specific contexts.

### *MiMIC* conversion utility and expansion potential

Taken together, we have developed and tested a new set of binary factor conversion cassettes that take advantage of the T2A polycistronic strategy to convert coding intron *MiMIC* insertions into reliable gene-specific *GAL4* and *GAL80* binary factors that can be used in a range of applications. This new strategy will significantly expand the utility of the growing number of publicly available *MiMIC* insertions because it more than triples the number of *MiMICs* that can be converted into reliable *GAL4* and *GAL80* binary factors.

Comparison of gene-trap and protein-trap binary factor conversions in the same locus and known expression patterns of some of the converted loci suggests that these new tools faithfully reflect the endogenous expression of the locus in which the *MiMIC* transposon is inserted, irrespective of its original orientation (i.e. the original transposon can be in the forward or reverse orientation, Figure 1a). It is important to note that insertions capturing all splice variants may be required to report the full expression pattern of the host gene and that some splice variants may not be separable. In this study we mostly used insertion sites that capture all transcript variants. The only exceptions were the 5′ non-coding and coding intronic *MiMIC* insertions in the 5-HT2A locus. However, these two 5-HT2A insertions together do capture all splice variants and produce very similar expression patterns. In theory, splice-variant specific insertions should nonetheless be usable to reveal expression of a subset of variants. This will depend on the saturation of the growing *MiMIC* library and may become an additional useful component of this strategy. Alternatively, *attP* sites could be introduced into specific introns of a gene using targeted CRISPR/cas9 nuclease strategies (40,41) to selectively capture-specific splice variants.

Similarly, CRISPR/cas9-based knock-in strategies could be used to introduce *attP* sites in specific genes in mammalian genomes. In combination with the strategy that we developed here, such an approach could then be used to generate allelic series in mammalian genes to better dissect expression, structure and function.

In addition to the novel protein-trap configuration tools, we created three additional binary factors that can be used to convert genes into non-overlapping or inducible binary factors. Together these new tools expand the repertoire and flexibility of the *MiMIC* transposon platform to allow further gene-specific manipulations such as expression pattern identification, expression-specific rescue experiments and manipulation of neuronal function (42–45). Given the large number of genes in the *Drosophila* genome that already contain *MiMIC* insertions in coding and non-coding introns, these new tools complement and improve upon the large collections of GAL4 lines generated by enhancer analysis (46), and should allow many investigators to better dissect the function of their genes of interest. Future incorporation of *in vivo* remobilization features would eliminate microinjection. Recently, the integrase swappable *in vivo* targeting element system was developed to convert different binary factors *in vivo* by using a vector with non-overlapping site-specific recombinase target sequences (47). Addition of these sequence elements to our conversion cassettes may allow expansion of the gene-specific approach presented here into a large-scale *in vivo* format.

## NOTE ADDED IN PROOF

While our manuscript was in the review process, two other manuscripts came to our attention describing a very similar technology (48) and an expanded version of the MiMIC library (49).

## SUPPLEMENTARY DATA

Supplementary Data are available at NAR Online.

SUPPLEMENTARY DATA
