# Supplementary material for: Gene-specific cell labeling using MiMIC transposons
Source: Nucleic Acids Res. 2015 Feb 20;43(8):e56. doi: 10.1093/nar/gkv113 (PMC4417149; doi:10.1093/nar/gkv113)
Supplement: SUPPLEMENTARY DATA [file supp_43_8_e56__index.html]

Gene-specific cell labeling using MiMIC transposons — SUPPLEMENTARY DATA 

# Gene-specific cell labeling using *MiMIC* transposons

## SUPPLEMENTARY DATA

**Files in this Data Supplement:**

- Supplementary Information
- Supplementary Video 1
- Supplementary Video 2
- Supplementary Video 3
- Supplementary Video 4
- Supplementary Video 5
