# Supplementary material for: Gene-specific cell labeling using MiMIC transposons
Source: Nucleic Acids Res. 2015 Feb 20;43(8):e56. doi: 10.1093/nar/gkv113 (PMC4417149; doi:10.1093/nar/gkv113)
Supplement: SUPPLEMENTARY DATA [file supp_gkv113_nar-03643-met-g-2014-File008.pdf]

Supplementary Information For:

Gene-specific cell labeling using *MiMIC* transposons

Joshua P. Gnerer[1], Koen J.T. Venken[2,3,4,5,8], and Herman A. Dierick[1,5,6,8]

Department of Molecular & Human Genetics[1], Verna and Marrs McLean Department of Biochemistry and Molecular Biology[2], Department of Pharmacology[3], Dan L. Ducan Cancer Center[4], Program in Integrative and Molecular Biomedical Sciences[5], Department of Neuroscience[6], Department of Pathology and Immunology[7], Program in Developmental Biology[8], Baylor College of Medicine, Houston, TX 77030

Correspondence should be addressed to K.J.T.V (Koen.Venken@bcm.edu) or H.A.D. (Dierick@bcm.edu)

Supplementary Table 1: Primers for molecular characterization of conversion events

Primer	Sequence 5' to 3'
Orientation-MiL-F	GCGTAAGCTACCTTAATCTCAAGAAGAG
Orientation-MiR-R	CGCGGCGTAATGTGATTTACTATCATAC
GAL4-F	AACTGTGCATCGTGCACCATC
GAL4-R	TGATGAGCTGCCGAGTCAATC
GS-F	TCTCCTAGAGACAGAAGCAGGCTG
GS-R	GCTCATCCAAGAATACTGAATGAG
GAL80-F	CACAACATTTGGTCACACAATC
GAL80-R	CATCTATCAGCTCTTGCTCTG
LexA-LV3-F	TTGAAGGTCATTATCAGGTCGATC
LexA-LV3-R	CAATACGTGCGACAACGACCTGAC
EGFPmultido-F	GGATGACGGCACCTACAAGAC
EGFPmultido-R	GTGGCTGTTGAAGTTGTACTC

List of all the primers used for evaluating conversion events with the different constructs used in the manuscript.

Supplementary Figure 1: T2A Binary Factor sequence files

T2A-GAL4

ggatccGAAGGACGCGGAAGCCTGTTGACGTGCGGAGATGTGGAAGAGAATCCAGGACCGATGAAGCTACT
GTCTTCTATCGAACAAGCATGCGATATTTGCCGACTTAAAAAGCTCAAGTGCTCCAAAGAAAAACCGAAGT
GCGCCAAGTGTCTGAAGAACAACTGGGAGTGTCGCTACTCTCCCAAAACCAAAAGGTCTCCGCTGACTAGG
GCACATCTGACAGAAGTGGAATCAAGGCTAGAAAGACTGGAACAGCTATTTCTACTGATTTTTCCTCGAGA
AGACCTTGACATGATTTTGAAAATGGATTCTTTACAGGATATAAAAGCATTGTTAACAGGATTATTTGTAC
AAGATAATGTGAATAAAGATGCCGTCACAGATAGATTGGCTTCAGTGGAGACTGATATGCCTCTAACATTG
AGACAGCATAGAATAAGTGCGACATCATCATCGGAAGAGAGTAGTAACAAAGGTCAAAGACAGTTGACTGT
ATCGATTGACTCGGCAGCTCATCATGATAACTCCACAATTCCGTTGGATTTTATGCCCAGGGATGCTCTTC
ATGGATTTGATTGGTCTGAAGAGGATGACATGTCGGATGGCTTGCCCTTCCTGAAAACGGACCCCAACAAT
AATGGGTTCTTTGGCGACGGTTCTCTCTTATGTATTCTTCGATCTATTGGCTTTAAACCGGAAAATTACAC
GAACTCTAACGTTAACAGGCTCCCGACCATGATTACGGATAGATACACGTTGGCTTCTAGATCCACAACAT
CCCGTTTACTTCAAAGTTATCTCAATAATTTTCACCCCTACTGCCCTATCGTGCACTCACCGACGCTAATG
ATGTTGTATAATAACCAGATTGAAATCGCGTCGAAGGATCAATGGCAAATCCTTTTTAACTGCATATTAGC
CATTGGAGCCTGGTGTATAGAGGGGGAATCTACTGATATAGATGTTTTTTACTATCAAAATGCTAAATCTC
ATTTGACGAGCAAGGTCTTCGAGTCAGGTTCCATAATTTTGGTGACAGCCCTACATCTTCTGTCGCGATAT
ACACAGTGGAGGCAGAAAACAAATACTAGCTATAATTTTCACAGCTTTTCCATAAGAATGGCCATATCATT
GGGCTTGAATAGGGACCTCCCCTCGTCCTTCAGTGATAGCAGCATTCTGGAACAAAGACGCCGAATTTGGT
GGTCTGTCTACTCTTGGGAGATCCAATTGTCCCTGCTTTATGGTCGATCCATCCAGCTTTCTCAGAATACA
ATCTCCTTCCCTTCTTCTGTCGACGATGTGCAGCGTACCACAACAGGTCCCACCATATATCATGGCATCAT
TGAAACAGCAAGGCTCTTACAAGTTTTCACAAAAATCTATGAACTAGACAAAACAGTAACTGCAGAAAAAA
GTCCTATATGTGCAAAAAAATGCTTGATGATTTGTAATGAGATTGAGGAGGTTTCGAGACAGGCACCAAAG
TTTTTACAAATGGATATTTCCACCACCGCTCTAACCAATTTGTTGAAGGAACACCCTTGGCTATCCTTTAC
AAGATTCGAACTGAAGTGGAAACAGTTGTCTCTTATCATTTATGTATTAAGAGATTTTTTCACTAATTTTA
CCCAGAAAAAGTCACAACTAGAACAGGATCAAAATGATCATCAAAGTTATGAAGTTAAACGATGCTCCATC
ATGTTAAGCGATGCAGCACAAAGAACTGTTATGTCTGTAAGTAGCTATATGGACAATCATAATGTCACCCC
ATATTTTGCCTGGAATTGTTCTTATTACTTGTTCAATGCAGTCCTAGTACCCATAAAGACTCTACTCTCAA
ACTCAAAATCGAATGCTGAGAATAACGAGACCGCACAATTATTACAACAAATTAACACTGTTCTGATGCTA
TTAAAAAAACTGGCCACTTTTAAAATCCAGACTTGTGAAAAATACATTCAAGTACTGGAAGAGGTATGTGC
GCCGTTTCTGTTATCACAGTGTGCAATCCCATTACCGCATATCAGTTATAACAATAGTAATGGTAGCGCCA
TTAAAAATATTGTCGGTTCTGCAACTATCGCCCAATACCCTACTCTTCCGGAGGAAAATGTCAACAATATC
AGTGTTAAATATGTTTCTCCTGGCTCAGTAGGGCCTTCACCTGTGCCATTGAAATCAGGAGCAAGTTTCAG
TGATCTAGTCAAGCTGTTATCTAACCGTCCACCCTCTCGTAACTCTCCAGTGACAATACCAAGAAGCACAC
CTTCGCATCGCTCAGTCACGCCTTTTCTAGGGCAACAGCAACAGCTGCAATCATTAGTGCCACTGACCCCG
TCTGCTTTGTTTGGTGGCGCCAATTTTAATCAAAGTGGGAATATTGCTGATAGCTCATTGTCCTTCACTTT
CACTAACAGTAGCAACGGTCCGAACCTCATAACAACTCAAACAAATTCTCAAGCGCTTTCACAACCAATTG
CCTCCTCTAACGTTCATGATAACTTCATGAATAATGAAATCACGGCTAGTAAAATTGATGATGGTAATAAT
TCAAAACCACTGTCACCTGGTTGGACGGACCAAACTGCGTATAACGCGTTTGGAATCACTACAGGGATGTT
TAATACCACTACAATGGATGATGTATATAACTATCTATTCGATGATGAAGATACCCCACCAAACCCAAAAA
AAGAGTAAaatgaatcgtagatactgaaaaaccccgcaagttcacttcaactgtgcatcgtgcaccatctc
aatttctttcatttatacatcgttttgccttcttttatgtaactatactcctctaagtttcaatcttggcc
atgtaacctctgatctatagaatttttaaatgactagaattaatgcccatcttttttttggacctaaatt
cttcatgaaaatatattacgagggcttattcagaagcttatcgataccgtcgactaaagccaaatagaaat
tattcagttctggcttaagtttttaaaagtgatattatttatttggttgtaaccaaccaaaagaatgtaaa
taactaatacataattatgttagttttaagttagcaacaaattgattttagctatattagctacttggtta
ataaatagaatatatttatttaaagataattcgtttttattgtcagggagtgagtttgcttaaaaactcgt
ttagatccactagttctagagcggccggatcc

T2A-GeneSwitch

ggatccGAAGGACGCGGAAGCCTGTTGACGTGCGGAGATGTGGAAGAGAATCCAGGACCGATGAAGCTACT
GTCTTCTATCGAACAAGCATGCGATATTTGCCGACTTAAAAAGCTCAAGTGCTCCAAAGAAAAACCGAAGT
GCGCCAAGTGTCTGAAGAACAACTGGGAGTGTCGCTACTCTCCCAAAACCAAAAGGTCTCCGCTGACTAGG
GCACATCTGACAGAAGTGGAATCAAGGCTAGAAAGACTGGAACAGCTATTTCTACTGATTTTTCCTCGAGA
AGACCTTGACATGATTTTGAAAATGGATTCTTTACAGGATATAAAAGCATTGTTAGAATTCCCGGGTGTCG
ACCAGAAAAAGTTCAATAAAGTCAGAGTTGTGAGAGCACTGGATGCTGTTGCTCTCCCACAGCCAGTGGGC

GTTCCAAATGAAAGCCAAGCCCTAAGCCAGAGATTCACTTTTTCACCAGGTCAAGACATACAGTTGATTCC
ACCACTGATCAACCTGTTAATGAGCATTGAACCAGATGTGATCTATGCAGGACATGACAACACAAAACCTG
ACACCTCCAGTTCTTTGCTGACAAGTCTTAATCAACTAGGCGAGAGGCAACTTCTTTCAGTAGTCAAGTGG
TCTAAATCATTGCCAGGTTTTCGAAACTTACATATTGATGACCAGATAACTCTCATTCAGTATTCTTGGAT
GAGCTTAATGGTGTTTGGTCTAGGATGGAGATCCTACAAACACGTCAGTGGGCAGATGCTGTATTTTGCAC
CTGATCTAATACTAAATGAACAGCGGATGAAAGAATCATCATTCTATTCATTATGCCTTACCATGTGGCAG
ATCCCACAGGAGTTTGTCAAGCTTCAAGTTAGCCAAGAAGAGTTCCTCTGTATGAAAGTATTGTTACTTCT
TAATACAATTCCTTTGGAAGGGCTACGAAGTCAAACCCAGTTTGAGGAGATGAGGTCAAGCTACATTAGAG
AGCTCATCAAGGCAATTGGTTTGAGGCAAAAAGGAGTTGTGTCGAGCTCACAGCGTTTCTATCAACTTACA
AAACTTCTTGATAACTTGCATGATCTTGTCAAACAACTTCATCTGTACTGCTTGAATACATTTATCCAGTC
CCGGGCACTGAGTGTTGAATTTCCAGAAATGATGTCTGAAGTTATTGCTGGGTCGACGAGATATCAAGCAG
AATTCCAGTACCTGCCAGATACAGACGATCGTCACCGGATTGAGGAGAAACGTAAAAGGACATATGAGACC
TTCAAGAGCATCATGAAGAAGAGTCCTTTCAGCGGACCCACCGACCCCCGGCCTCCACCTCGACGCATTGC
TGTGCCTTCCCGCAGCTCAGCTTCTGTCCCCAAGCCAGCACCCCAGCCCTATCCCTTTACGTCATCCCTGA
GCACCATCAACTATGATGAGTTTCCCACCATGGTGTTTCCTTCTGGGCAGATCAGCCAGGCCTCGGCCTTG
GCCCCGGCCCCTCCCCAAGTCCTGCCCCAGGCTCCAGCCCCTGCCCCTGCTCCAGCCATGGTATCAGCTCT
GGCCCAGGCCCCAGCCCCTGTCCCAGTCCTAGCCCCAGGCCCTCCTCAGGCTGTGGCCCCACCTGCCCCCA
AGCCCACCCAGGCTGGGGAAGGAACGCTGTCAGAGGCCCTGCTGCAGCTGCAGTTTGATGATGAAGACCTG
GGGGCCTTGCTTGGCAACAGCACAGACCCAGCTGTGTTCACAGACCTGGCATCCGTCGACAACTCCGAGTT
TCAGCAGCTGCTGAACCAGGGCATACCTGTGGCCCCCCACACAACTGAGCCCATGCTGATGGAGTACCCTG
AGGCTATAACTCGCCTAGTGACAGGGGCCCAGAGGCCCCCCGACCCAGCTCCTGCTCCACTGGGGGCCCCG
GGGCTCCCCAATGGCCTCCTTTCAGGAGATGAAGACTTCTCCTCCATTGCGGACATGGACTTCTCAGCCCT
GCTGAGTCAGATCAGCTCCTAAggggtgacgcctgccctccccagagcactgggttgcaggggattgaag
ccctccaaaagcacttacggattctggtggggtgtgttccaactgcccccaactttgtggatgtcttcctt
ggaggggggagccatattttattcttttattgtcagtatctgtatctctctctctttttggaggtgcttaa
gcagaagcattaacttctctggaaagggggagctggggaaactcaaacttttccccctgtcctgatggtca
gctcccttctctgtagggaactctggggtcccccatccccatcctccagcttctggtactctcctagagac
agaagcaggctggaggtaaggcctttgagcccacaaagccttatcaagtgtcttccatcatggattcatta
cagcttaatcaaaataacgccccagataccagcccctgtatggcactggcattgtccctgtgcctaacacc
agcgtttgaggggctggccttcctgccctacagaggtctctgccggctctttccttgctcaaccatggctg
aaggaaaccagtgcaacagcactggctctctccaggatccgacgcgtcgagcttatcgataccgtcgacta
aagccaaatagaaattattcagttctggcttaagtttttaaaagtgatattatttatttggttgtaaccaa
ccaaaagaatgtaaataactaatacataattatgttagttttaagttagcaacaaattgattttagctata
ttagctacttggttaataaatagaatatatttatttaaagataattcgtttttattgtcagggagtgagtt
tgcttaaaaactcgtttagatccactagttctagagcggcc**ggatcc**

T2A-LexA

ggatccGAAGGACGCGGAAGCCTGTTGACGTGCGGAGATGTGGAAGAGAATCCAGGA**CCGATGAAAGCGTT**
AACGGCCAGGCAACAAGAGGTGTTTGATCTCATCCGTGATCACATCAGCCAGACAGGTATGCCGCCGACGC
GTGCGGAAATCGCGCAGCGTTTGGGGTTCCGTTCCCCAAACGCGGCTGAAGAACATCTGAAGGCGCTGGCA
CGCAAAGGCGTTATTGAAATTGTTTCCGGCGCATCACGCGGGATTCGTCTGTTGCAGGAAGAGGAAGAAGG
GTTGCCGCTGGTAGGTCGTGTGGCTGCCGGTGAACCACTTCTGGCGCAACAGCATATTGAAGGTCATTATC
AGGTCGATCCTTCCTTATTCAAGCCGAATGCTGATTTCCTGCTGCGCGTCAGCGGGATGTCGATGAAAGAT
ATCGGCATTATGGATGGTGACTTGCTGGCAGTGCATAAAACTCAGGATGTACGTAACGGTCAGGTCGTTGT
CGCACGTATTGATGACGAGGTTACCGTTAAGCGCCTGAAAAAACAGGGCAATAAAGTCGAACTGTTGCCAG
AAAATAGCGAGTTTAAACCAATTGTCGTAGATCTTCGTCAGCAGAGCTTCACCATTGAAGGGCTGGCGGTT
GGGGTTATTCGCAACGGCGACTGGCTGGGATCTCCGGCCGACGCCCTGGACGACTTCGACCTGGACATGCT
GCCGGCCGACGCCCTGGACGACTTCGACCTGGACATGCTGCCGGCCGACGCCCTGGACGACTTCGACCTGG
ACATGCTGCCGGGGTAActaagtaaggatctagacatgataagatacattgatgagtttggacaaagctta
tcgataccgtcgactaaagccaaatagaaaattattcagttcctggcttaagtttttaaaagtgatattat
ttatttggttgtaaccaaccaaaagaatgtaaataactaatacataattatgttagttttaagttagcaac
aaattgattttagctatattagctacttggttaataaatagaatatatttatttaaagataattgcgtttt
tattgtcagggagtgagtttgcttaaaaactcgtttagatccactagttctagagcggcc**ggatcc**

T2A-GAL80

ggatccGAAGGACGCGGAAGCCTGTTGACGTGCGGAGATGTGGAAGAGAATCCAGGA**CCGATGGACTACAA**
CAAGAGATCTTCGGTCTCAACCGTGCCTAATGCAGCTCCCATAAGAGTCGGATTCGTCGGTCTCAACGCAG

CCAAAGGATGGGCAATCAAGACACATTACCCCGCCATACTGCAACTATCGTCACAATTTCAAATCACTGCC
TTATACAGTCCAAAAATTGAGACTTCTATTGCCACCATCCAGCGTCTAAAATTGAGTAATGCCACTGCTTT
TCCCACTTTAGAGTCATTTGCATCATCTTCCACTATAGATATGATAGTGATAGCTATCCAAGTGGCCAGCC
ATTATGACGTTGTTATGCCTCTCTTGGAATTCTCCAAAAATAATCCGAACCTCAAGTATCTTTTCGTAGAA
TGGGCCCTTGCATGTTCACTAGATCAAGCCGAATCCATTTATAAGGCTGCTGCTGAACGTGGGGTTCAAAC
CATCATCTCTTTACAAGGTCGTAAATCACCATATATTTTGAGAGCAAAAGAATTAATATCTCAAGGCTATA
TCGGCGACATTAATTCGATCGAGATTGCTGGAAATGGCGGTTGGTACGGCTACGAAAGGCCTGTTAAATCA
CCAAAATACATCTATGAAATCGGGAACGGTGTAGATCTGGTAACCACAACATTTGGTCACACAATCGATAT
TTTACAATACATGACAAGTTCGTACTTTTCCAGGATAAATGCAATGGTTTTCAATAATATTCCAGAGCAAG
AGCTGATAGATGAGCGTGGTAACCGATTGGGCCAGCGAGTCCCAAAGACAGTACCGGATCATCTTTTATTC
CAAGGCACATTGTTAAATGGCAATGTTCCAGTGTCATGCAGTTTCAAAGGTGGCAAACCTACCAAAAAATT
TACCAAAAATTTGGTCATTGACATTCACGGTACCAAGGGAGATTTGAAACTTGAAGGCGATGCCGGATTCG
CAGAAATTTCAAATCTGGTCCTTTACTACAGTGGAACTAGAGCAAACGACTTCCCGCTAGCCAATGGACAA
CAAGCTCCTTTAGACCCGGGGTATGATGCAGGTAAAGAAATCATGGAAGTATATCATTTACGAAATTATAA
TGCCATTGTGGGTAACATTCATCGACTGTATCAATCTATCTCTGACTTCCACTTCAATACAAAGAAAATTC
CTGAATTACCCTCACAATTTGTAATGCAAGGTTTCGATTTCGAAGGCTTTCCCACCTTGATGGATGCTCTG
ATATTACACAGGTTAATCGAGAGCGTTTATAAAAGTAACATGATGGGCTCCACATTAAACGTTAGCAATAT
CTCGCATTATAGTTTATAAatgaatcgtttttaaaataacaaatcaattgttttataatattcgtacgatt
ctttgattatgtaataaaatgtgatcattaggaagattacgaaaaatataaaaaatatgagttctgtgtgt
ataacaaatgctgtaaacgccacaattgtgtttgttgcaaataaacccatgattatttgattaaaattgtt
gttttctttgttcatagacaatagtgtgttttgcctaaacgtgtactgcataaactccatgcgagtgtata
gcgagctagtggctaacgcttgccccaccaaagtagattcgtcaaaatcctcaatttcatcaccctcctcc
aagtttaacatttggccgtcggaattaacttctaaagatgccacataatctaataaatgaaatagagattc
aaacgtggcgtcatcgtccgtttcgaccatttccgaaaagaactcgggcataaactctatgatttctctgg
acgtggtgttgtcgaaactctcaaagtacgcagtcaggaacgtgcgcgacatgtcgtcgggaaactcgcgc
ggaaacatgttgttgtaaccgaacgggtcccatagcgccaaaaccaaatctgccagcgtcaatagaatgag
cacgatgccgacaatggagctggcttggatagcgattcgagttaac**ggatcc**

Legend: Green caps: T2A tag
Blue caps: GAL4, GS, LexA, GAL80 ORFs
*Bam*HI cloning sites are bold faced at the 5' and 3' ends

Sequences of the different *T2A*-binary factor constructs that were generated for this study. Primers used for the cloning strategy are shown as underlined sequence at the 5' and 3' ends of the sequence files. Forward primers also included the *T2A* sequence and *Bam*HI site. The *GeneSwitch* constructs were cloned by swapping the *Xho*I-*Spe*I fragment (restriction sites are marked in red) from the *T2A-GAL4* constructs and replacing it with a *Xho*I-*Spe*I fragment from the GeneSwitch vector (pP{w/lo GS}). Underlined red colored codons in *T2A-GAL80* are polymorphic sequences compared to the published *GAL80* sequence. Underlined sequences correspond to the primer sequences used to sequence the cloned constructs.

<u>Supplementary Figure 2</u>: Locus maps of all the genes and alleles used in this manuscript

a. *5-HT1A* locus allele map

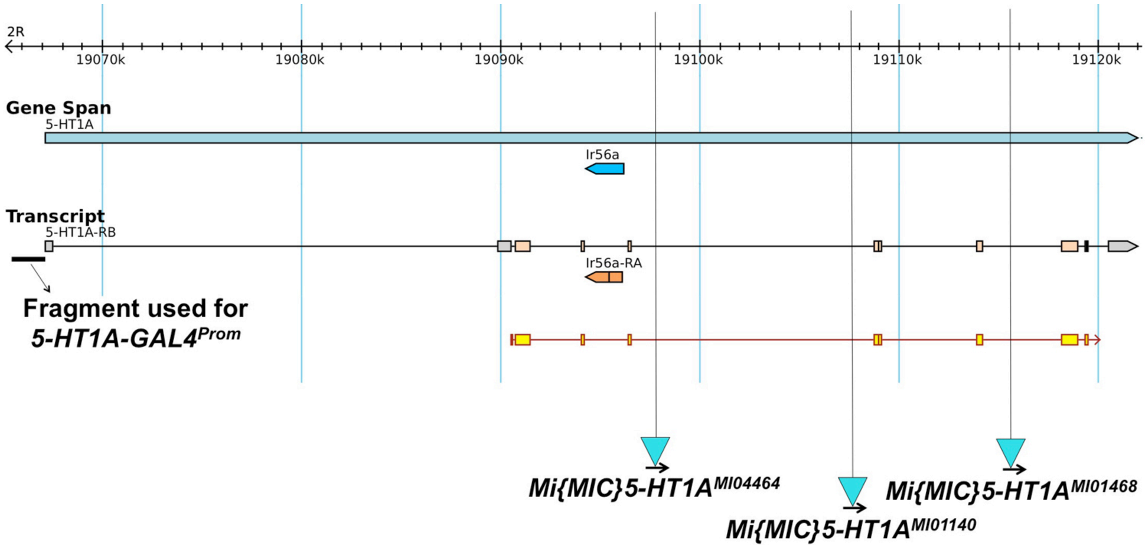

b. *5-HT1B* locus allele map

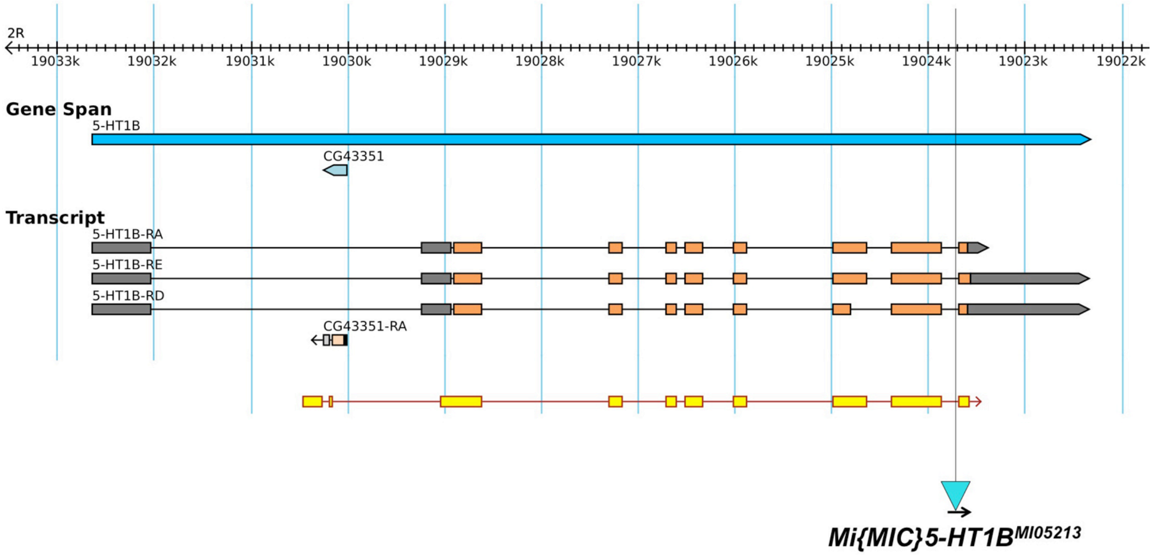

c. *5-HT2A* locus allele map

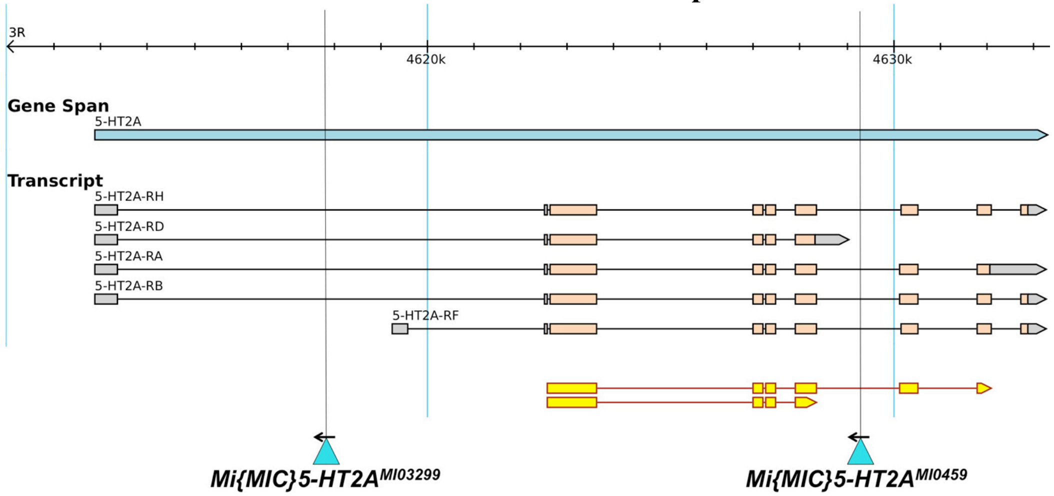

d. *5-HT2B* locus allele map

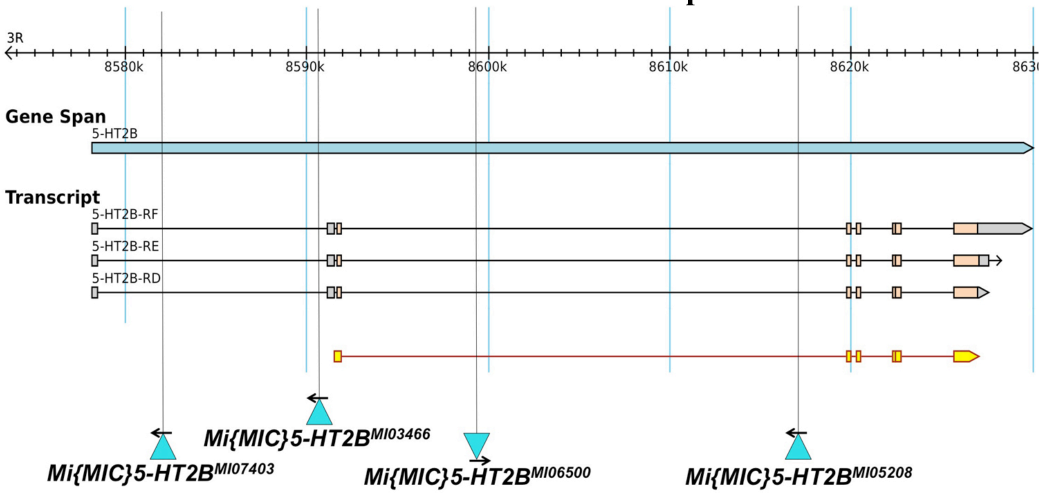

e. *5-HT7* locus allele map

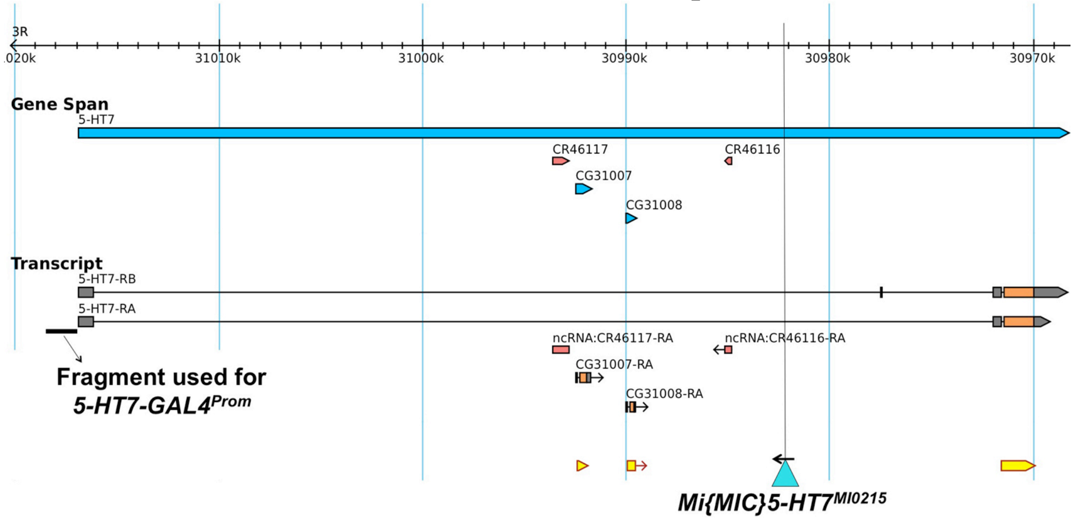

f. *arm* locus allele map

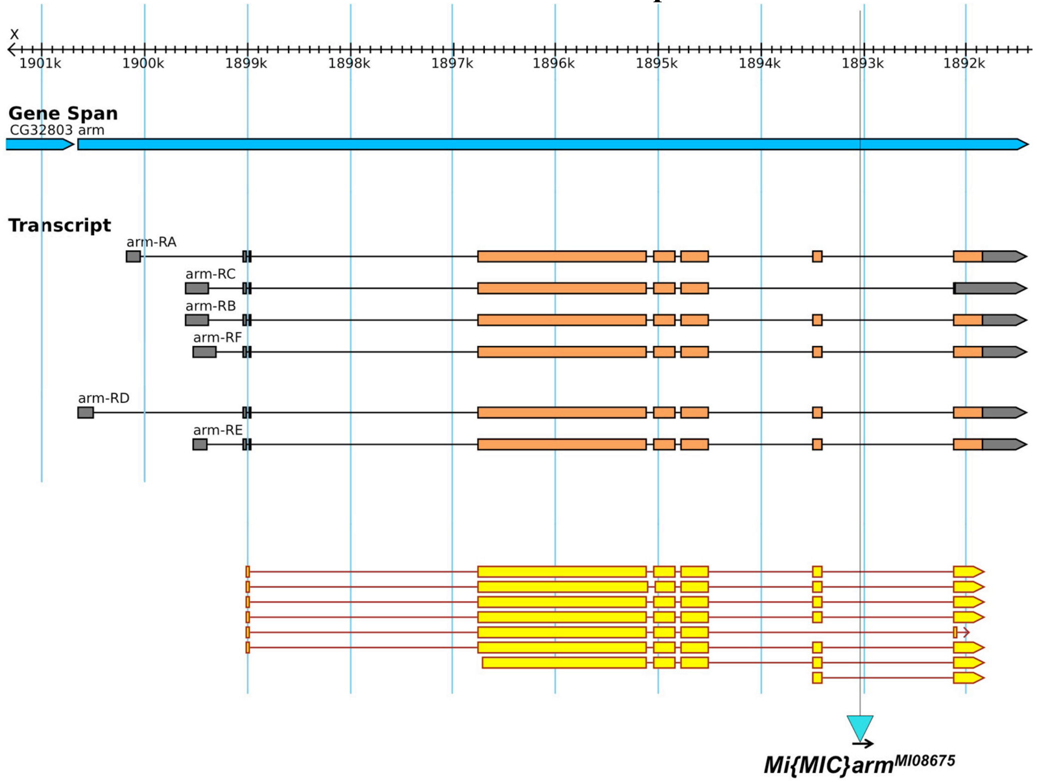

g. *Vmat* locus allele map

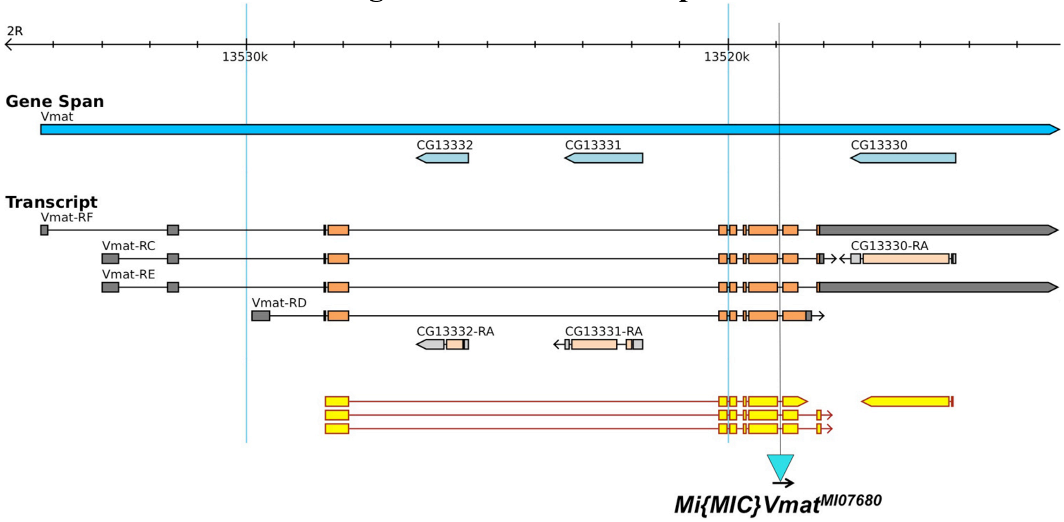

h. *Ubp64E* locus allele map

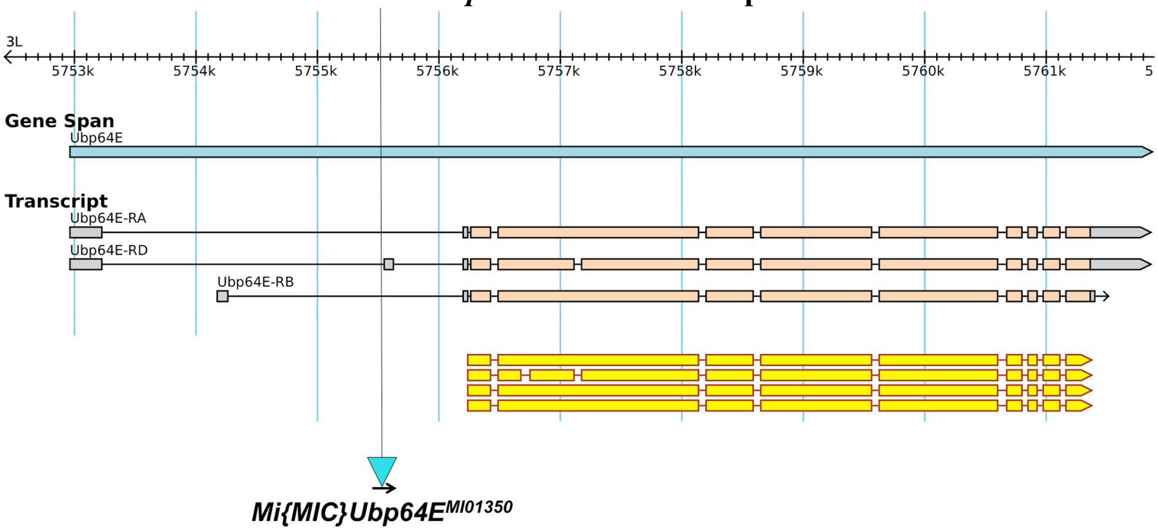

i. *gfA* locus allele map

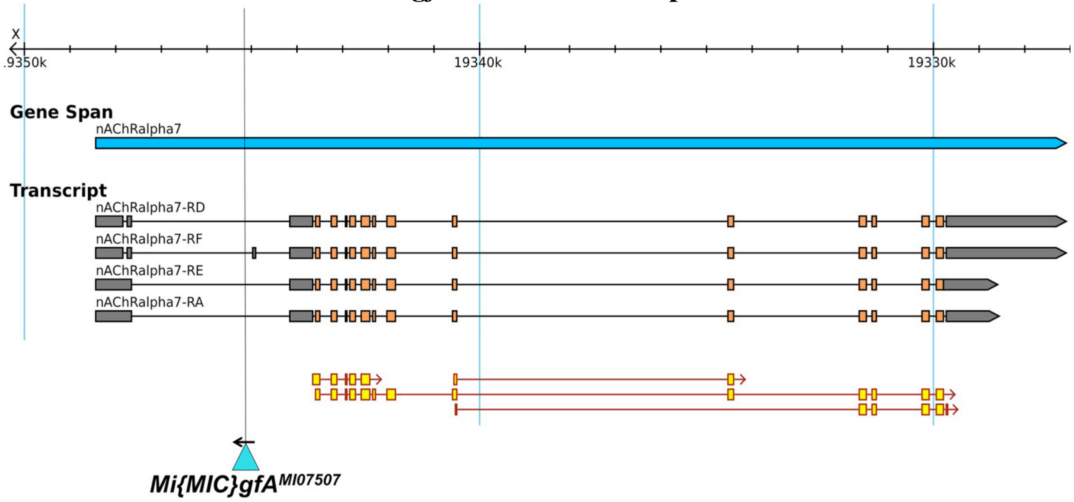

j. *Itp* locus allele map

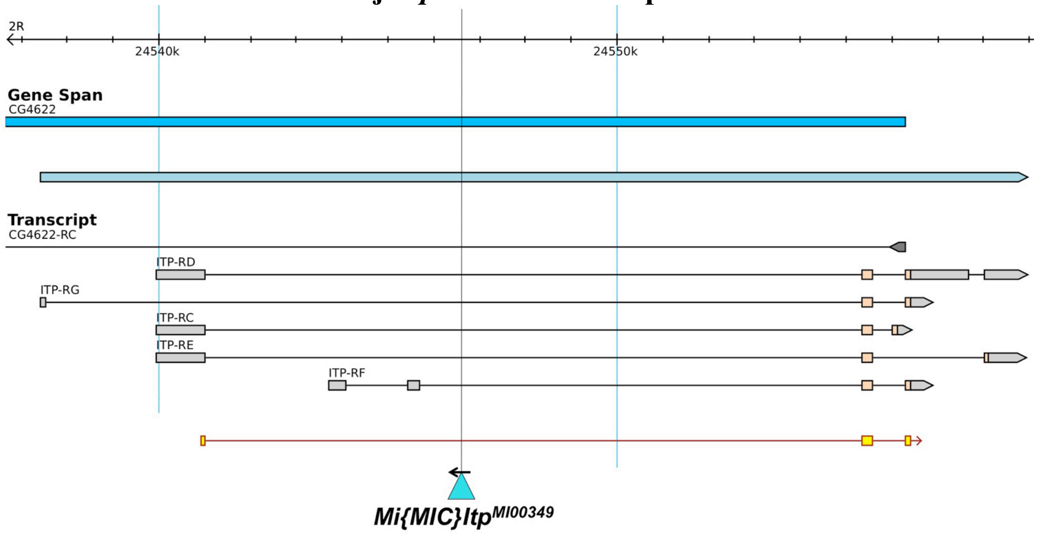

Locus maps of all the genes and alleles used in this manuscript. All genes are shown in the 5’ to 3’ direction regardless of the strand from which they are expressed in the *Drosophila* genome. *MiMIC* insertions are shown as a vertical line ending in a blue triangle and are annotated by their *MI* number. The orientation of the insertion with respect to the transcriptional direction of the gene is shown with an arrow below the blue triangle.

<u>Supplementary Figure 3</u>: Expression patterns of different *MiMIC* insertions in *5-HT2B*

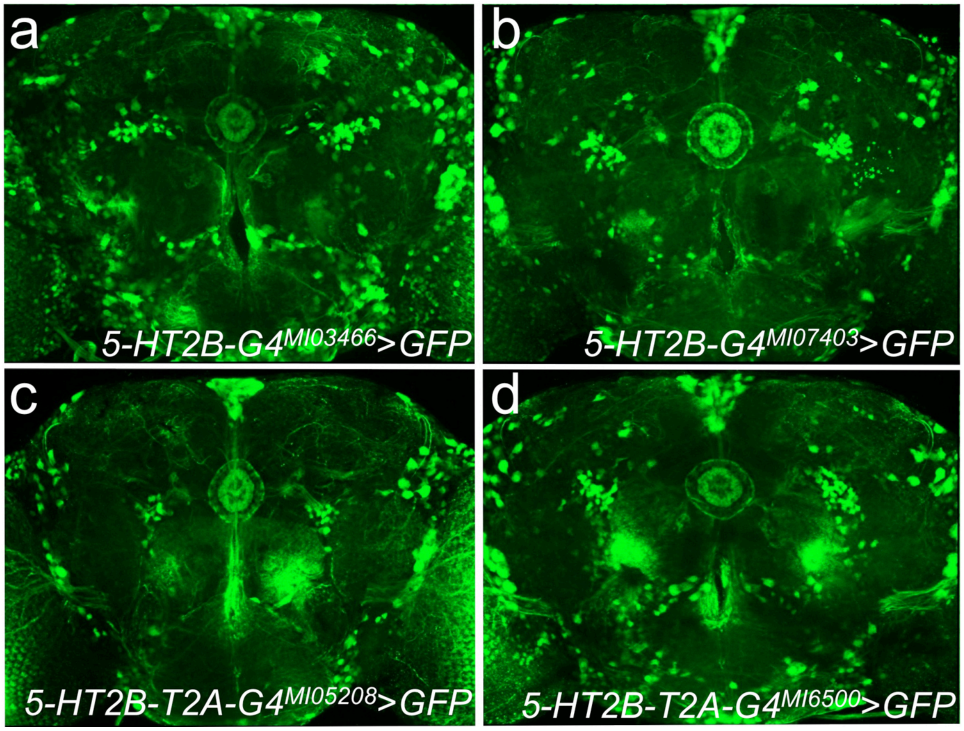

(**a-d**) GFP staining pattern of the four gene-trap and protein-trap conversions in the *5-HT2B* locus (*Mi{MIC}5-HT2B-T2A-GAL4*^{MI03466}, *Mi{MIC}5-HT2B-T2A-GAL4*^{MI03466} , *Mi{MIC}5-HT2B-T2A-GAL4*^{MI07403}, *Mi{MIC}5-HT2B-T2A-GAL4*^{MI05208}, and *Mi{MIC}5-HT2B-T2A-GAL4*^{MI06500}) driving expression of *UAS-GFP*. All conversion alleles show strong expression in *PI* neurons, EB, LTR and R-cells as well as scattered neurons throughout the protocerebrum. The patterns are very similar. *PI*, *pars intercerebralis*; EB, ellipsoid body; LTR, lateral triangle; R-cells, neuron ring neurons of the EB.

<u>Supplementary Figure 4</u>: Comparison of EGFP protein-trap with T2A-GAL4 protein-trap of Armadillo

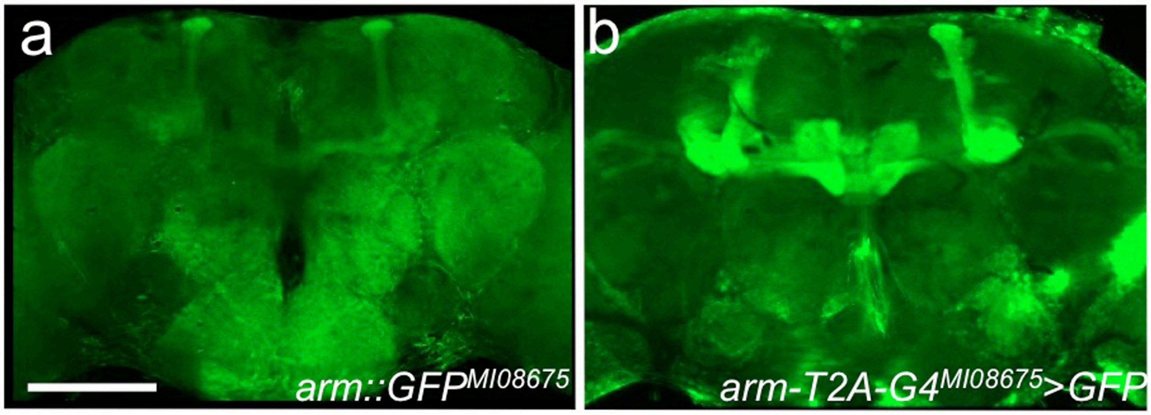

(a) An internally tagged EGFP protein fusion of *arm* (*arm::EGFP*^{MI08675}) shows neuropil staining throughout the brain including the MBs. (**b**) A protein-trap event between arm and T2A-GAL4 (*arm-T2A-GAL4*^{MI08675}) driving the expression of *UAS-GFP* also shows broad neuropil staining with stronger expression in the MBs.

<u>Supplementary Video 1</u>: 3-D reconstruction of *Mi{MIC}5-HT1A-T2A-GAL4*^{MI01140} expression pattern.

<u>Supplementary Video 2</u>: 3-D reconstruction of *Mi{MIC}5-HT1B-T2A-GAL4*^{MI05213} expression pattern.

<u>Supplementary Video 3</u>: 3-D reconstruction of *Mi{MIC}5-HT2A-GAL4*^{MI03299} expression pattern.

<u>Supplementary Video 4</u>: 3-D reconstruction of *Mi{MIC}gfA-GAL4*^{MI07507} expression pattern.

<u>Supplementary Video 5</u>: 3-D reconstruction of *Mi{MIC}ITP-GAL4*^{MI00349} expression pattern.
